# Effectiveness of Non‐Pharmacological Therapies on Behavioral Symptoms and Cognitive Functioning of Low‐Risk Nursing Home Patients

**DOI:** 10.1002/alz70858_103858

**Published:** 2025-12-25

**Authors:** Reena Sethi, Ihita Bolisetti, Rhea Chatterjee, Punav Khetarpal, Riya Bajpai, Olivia LePage, Aditri Balaji, Kaitlyn Baker

**Affiliations:** ^1^ Virginia Commonwealth University, Richmond, VA, USA

## Abstract

**Background:**

As the global aging population is estimated to double from 2015 to 2050, it has become increasingly critical to address cognitive and behavioral decline in elderly populations—particularly those living in nursing home facilities. In this review, we aim to holistically assess the effectiveness of routine art‐based activities and mentally stimulating tasks as interventions for addressing depression and cognitive deterioration prevalent in nursing homes.

**Method:**

The literature reviewed employed a randomized controlled trial in which nursing home residents were divided into an art‐based therapy group (painting, and drawing), mentally stimulating task groups (basic arithmetic, reading comprehension, and memory games), as well as a control group. Sessions were conducted three to five times per week consisting of 60‐120 minutes of engaged activity. Cognitive function was measured using the Mini‐mental State Examination (MMSE), the Frontal Assessment Battery (FAB), and the Geriatric Depression Scale (GDS). Surveys were utilized to gauge perceived benefits.

**Results:**

Multiple cognitive and emotional assessments from this cross‐literature review revealed the prominence of loneliness and limited mental stimulation in nursing home residents, often exacerbated by neglect and resource limitations. Structured interventions such as art therapy showed improved mood, boosted self‐esteem, and reduced loneliness and depression. Intellectually stimulating tasks like arithmetic and reading revealed heightened cognitive functioning, particularly in memory, attention, and executive functioning.

**Conclusion:**

This literature review highlights the value of structured and interactive non‐pharmacological interventions in improving social engagement, cognitive functioning, and emotional well‐being in nursing home residents.

